# De novo characterization of venom apparatus transcriptome of *Pardosa pseudoannulata* and analysis of its gene expression in response to Bt protein

**DOI:** 10.1186/s12896-017-0392-z

**Published:** 2017-11-07

**Authors:** Rong Li, Zhenzhen Yan, Juan Wang, Qisheng Song, Zhi Wang

**Affiliations:** 1 College of Bioscience & Biotechnology, Hunan Agriculture University, Changsha, 410128 China; 2 Department of Biosciences, Hunan University of Arts and Science, Changde, 415000 China; 3 Division of Plant Sciences, University of Missouri, Columbia, MO 65211 USA

**Keywords:** The venom apparatus, Transcriptome, *Pardosa pseudoannulata*, Bt protein, Immune system

## Abstract

**Background:**

*Pardosa pseudoannulata* is a prevailing spider species, and has been regarded as an important bio-control agent of insect pests in farmland of China. However, the available genomic and transcriptomic databases of *P. pseudoannulata* and their venom are limited, which severely hampers functional genomic analysis of *P. pseudoannulata*. Recently high-throughput sequencing technology has been proved to be an efficient tool for profiling the transcriptome of relevant non-target organisms exposed to *Bacillus thuringiensis* (Bt) protein through food webs.

**Results:**

In this study, the transcriptome of the venom apparatus was analyzed. A total of 113,358 non-redundant unigenes were yielded, among which 34,041 unigenes with complete or various length encoding regions were assigned biological function annotations and annotated with gene ontology and karyotic orthologous group terms. In addition, 3726 unigenes involved in response to stimulus and 720 unigenes associated with immune-response pathways were identified. Furthermore, we investigated transcriptomic changes in the venom apparatus using tag-based DGE technique. A total of 1724 differentially expressed genes (DEGs) were detected, while 75 and 372 DEGs were functionally annotated with KEGG pathways and GO terms, respectively. qPCR analyses were performed to verify the DEGs directly or indirectly related to immune and stress responses, including genes encoding heat shock protein, toll-like receptor, GST and NADH dehydrogenase.

**Conclusion:**

This is the first study conducted to specifically investigate the venom apparatus of *P. pseudoannulata* in response to Bt protein exposure through tritrophic chain. A substantial fraction of transcript sequences was generated by high-throughput sequencing of the venom apparatus of *P. pseudoannulata*. Then a comparative transcriptome analysis showing a large number of candidate genes involved in immune response were identified by the tag-based DGE technology. This transcriptome dataset will provide a comprehensive sequence resource for furture molecular genetic research of the venom apparatus of *P. pseudoannulata*.

**Electronic supplementary material:**

The online version of this article (10.1186/s12896-017-0392-z) contains supplementary material, which is available to authorized users.

## Background

Animals use the venom apparatus for defense and predation by injecting their venomous mixture into their preys, and venom also have important biological implications with biomedical and agricultural relevance [1]. Only certain animals synthesize venom, but their production are taxonomically widespread in invertebrates and vertebrates because they have evolved independently on numerous occasions [2]. Venom genes have already been previously reported in snakes, scorpions, stingrays and parasitic wasps [3–6]. Spider venoms recently gathered far more attention from worldwide research groups [7]. Because spider venoms are complex mixture, mainly containing disulfide peptides that typically have high affinity and specificity for particular subtypes of ion channels and receptors, they can be used as leads for developing therapeutics and bioinsecticides [8, 9].

However, our understanding of spiders and their venom is limited, due in large part to the lack of spider genome database and comprehensive transcriptome dataset [6]. With the exceedingly rapid development of next-generation sequencing technology (NGS) and drop in cost, the number of sequencing applications are increasing exponentially [10].

It is widely known that *Bacillus thuringiensis* (*Bt*) strains can produce Cry proteins that posses insecticidal activity and induce the formation of pores in midgut membranes of susceptible organisms. Therefore, Bt proteins have been employed as bioinsecticides against target organisms, such as lepidopteran pest larvae [11]. However, the possibility of adverse impacts on the non-target species remains a concern. In fact, many researchers are specialized in elucidating the mechanism of the effect of Bt proteins on non-target organisms via tritrophic food chain [12, 13]. Non-target organisms involved in these recent researches can be generally classified into two categories: model and non-model organisms. For example, Tang et al. conducted a research that when Sprague Dawley rats were fed with the rice line T1C-1 expressing Cry1C for 90 days, this Bt rice line had no influence on rat behavior, weight gain and blood biochemical parameters. At the same time, no histopathological changes were recorded [14]. In a related investigation of other model organism, zebrafish *Danio rerio,* it was found that Bt maize is just as safe and nutritious as non-Bt maize for two generations of zebrafish when they were fed with casein/gelatin-based diets containing 19% Bt maize [15]. With regard to the non-model organisms, on the one hand, studies usually centered on the effect of Bt protein on its ecological behavior, for example, food consumption of *Apis mellifera* [16] or growth and predation of *Hylyphantes graminicola* (Araneae: Linyphiidae) and *Coleosoma octomaculatum* (Araneae: Theridiidae) [17] or survival and fecundity of *Daphnia magna* exposed to the Bt maize [18]. On the other hand, some searchers have obtained massive amounts of sequence data for target organisms using NGS, but these profiles did not cover the effect of Bt protein on non-target organisms and their apparatus. For instance, transcriptomes of three orb-web spiders (*Gasteracantha arcuata, Nasoonaria sinensis and Gasteracantha hasselti*) were analyzed for revealing gene expression profiles associated with toxin and silk [19], but transcriptomic analysis of venom glands was performed only for a single fishing spider *Dolomedes mizhoanus* [20]. A series of studies of Bt effect on *P. pseudoannulata* have been performed in our lab. One of our studies showed that developmental time of the spiders from the 2nd to 8th instars was affected by Bt rice. Another study also showed that the activities of three key metabolic enzymes, acetylcholine esterase (AchE), glutathione peroxidase (GSH-Px), and superoxide dismutase (SOD) were significantly influenced in the spiders after feeding on Cry1Ab-containing fruit flies [21, 22]. Nevertheless, the Bt effect on the gene expression in venom glands of spiders has not been reported. In the study, we wanted to explore whether Bt protein has any impact on the venom apparatus of *P. pseudoannulata* through food webs using transcriptome analysis.

Up to now, only *Tetranychus urticae* (the two-spotted spider mite) genome and *Ixodes scapularis* (blacklegged tick) genome were sequenced in the class Arachnida, phylum Arthropoda [23]. Many transcriptomes of arachnida were analyzed using Illumina sequencing, including four suborders of harvestmen [24], *Tetranychus cinnabarinus* [25], *Theridion grallator*, *T. californicum* [26] and so on. In recent years, more studies were focused on the transcriptomes of apparatus, for example, venom glands of *Hadrurus gertschi* [27], *Latrodectus tredecimguttatus* [28], spinning glands of *Actinopus spp.* and *Gasteracantha cancriformis* [29], salivary glands of *Ixodes ricinus* [30], and ovaris of *Rhipicephalus (Boophilus) microplus* [31]. Comparing with conventional sequencing methods, the NGS provides a more ideal method for the analysis of transcriptome with high efficiency, and facilitates the studies on arachnidas’ gene background.

In this paper, a global genetic and transcriptome-based analysis of the venom apparatus of *P. pseudoannulata* [30, 32] exposed to the Bt protein by tritrophic pathway was examined using high throughput next-generation RNA-sequencing technique coupled with bioinformatics. In addition, NGS technology was applied to analyze the differentially expressed genes (DEGs) in the venom apparatus of *P. pseudoannulata* following a tritrophic model: the wolf spiders preyed on brown planthoppers (BPHs) *Nilaparvata lugens*, which were reared on the Cry1Ab-expressing rice or non-transgenic parental commercial cultivar. DEGs were further validated by qPCR analyses to gain insight into the response of venom apparatus to Bt protein exposure and to identify the genes that may respond to environmental stress or immunity andcould serve as targets for improved bioinsecticides.

## Methods

### Insect rearing and sample preparation

BPHs were gathered from Bt and non-Bt control rice after feeding on rice stems for 15 days, and used as foods for spiders. Approximately 70,000 BPHs were collected from each group [33].

Forty healthy *P. pseudoannulata* adults of similar size were collected from traditional fields in the experimental farmland of Hunan Academy of Agriculture Sciences (113.08°E, 28.18°N) in 2014. Each spider was transferred to a glass tube (15 mm diameter, 100 mm height) with moisten cotton ball covered at the bottom for water. The spiders were starved for 48 h and then fed with 15 Cry1Ab-containing BPHs (the average amount of Cry1Ab protein in BHPs: 0.47 ng/mg) or 15 non-Cry1Ab-containing BPHs every day. The feeding process was conducted in a temperature-controlled chamber (25 ± 2 °C, 70 ± 10% RH and L:D 16:8 photoperiod, photophase from 06:00 to 22:00).

### RNA isolation

This work was done by Oebiotech Enterprise, Shanghai. The venom apparatus (containing main gland, venom canal and fang) from a total of 19 control and 21 Bt-treated spiders, respectively, were carefully separated from other tissues, rinsed thoroughly in phosphate-buffer saline (PBS) solution (pH 7.2) to remove debris, and pooled. Total RNA of each sample was extracted immediately upon dissection using TRIzol reagent (Invitrogen, USA) and used to generate cDNA library. The procedures were performed according to the manufacturer’s protocol. The quality and quantity of RNA were analyzed with electrophoresis in 1% TAE agarose gel and with a NanoDrop spectrophotometer (ThermoScientific, USA), respectively. The total RNAs with absorbance 260/280 nm ratio of 1.8~2.1 were chosen and treated with DNase I (Fermentas, Lithuania) prior to library construction. RNA samples were stored frozen at −80 °C until needed.

### cDNA library construction

Poly-(A) mRNA was purified using oligo (dT) magnetic beads and used for the first-strand cDNA synthesis with random hexamer-primer and reverse transcriptase (Invitrogen, USA). The second-strand cDNA was synthesized using dNTPs, buffer, RNaseH (Invitrogen, USA) and DNA polymerase I (TaKaRa, Japan). The double-strand cDNA was end-repaired using T4 DNA polymerase, Klenow fragment, and T4 polynucleotide kinase (Thermo Scientific, USA) followed by a single (A) base addition using Klenow 3′ to 5′ exo-ploymerase, and further ligated with PE Adapter Oligo Mix supplied by the mRNA-Seq Sample Preparation Kit (Illumina, USA) using T4 DNA ligase (Thermo Scientific, USA). The resulting cDNA with adaptor-ligated was then subjected to PCR amplification for about 15 cycles.

### Bioinformatics analysis of RNA-seq results

The cDNA library was sequenced on the Illmina Hiseq2000 platform. Sequencing-received raw image data were transformed by base calling into sequence data, which were called raw reads [34]. The raw reads as the direct product from Illmina sequencing were transformed into clean reads by removing low quality reads, adaptor sequence, fragments with bases N and duplication sequence. Then clean reads were de novo assembled with the short reads assembling program Trinity, which is a very favorable tool, especially in the absence of a reference genome [35]. The resulting unigenes with a minimal length of 200 bp were used for sequence similarity searches in the Nr/NCBI (non-redundant, http://www.ncbi.nlm.nih.gov), Swiss-Prot/UniProtKB (http://www.uniprot.org), KOG/NCBI (http://www.ncbi.nlm.nih.gov), KEGG (http://www.genome.jp/kegg/) and GO (http://geneontology.org/) with a siginificant threshold of E-value < 10^−5^.

### Analysis of differentially expressed genes (DEGs)

Gene expression of all unigenes in the venom apparatus of spiders fed with Bt BPHs and non-Bt BPHs were estimated by calculating read density as ‘reads per kilobase of exon model per million mapped reads’ (FPKM) [36]. In the present study, a Perl script was used to calculate FPKM for each assembled cDNA sequence with parsing alignment data using the software Bowtie 2 [37]. And we used the false discovery rate (FDR) method mentioned by Audic and Claverie to determine the *P*-value threshold for multiple testing by controlling the FDR value [38]. A threshold value of FDR <0.05 and absolute value of log2 fold change > 2 were used to identify DEGs.

### Validation of gene expression by qPCR

qPCR analysis was conducted to validate key venom apparatus genes of interest, which was identified either by their exhibition of relatively large expression fold-changes from DEG analyses, or by scientific literature about potential gene functions related to immunity. Total RNA of each sample was isolated with TRIzol (Invitrogen Corp, USA) and treated with DNase I (Fermentas, Lithuania) according to the manufacturer’s protocol. The cDNA was synthesized using a RevertAid™ H Minus First Strand cDNA Synthesis Kit (Fermentas, Lithuania) and the qRT-PCR was performed using ABI 7900HT (ABI, USA) with a volume of 25 μL, containing 1 μL of 1:10 cDNA diluted with ddH_2_O, 12.5 μL 2xSYBR Green Master Mix (ABI, USA) and 200 nM primer pair. Reactions were performed in triplicate to ensure consistent technical replication and run in 96-well plates under the following conditions: 94 °C for 3 min to activate the polymerase, then followed by 40 cycles for 94 °C for 30 s, 59 °C for 30 s, 72 °C for 45 s. Cycling ended at 72 °C for 5 min. Relative gene expression was evaluated with a DataAssist Software version 3.0 (Applied Biosystems/Life Technologies), using 18 s rRNA (primers: 5′- AGATGCCCTTAGATGTCCGG-3′ and 5′-AAGGGCAGGGACGTAATCAA-3′) as an endogenous control for RNA load and gene expression in analyses. The relative quantitative method (△△C_T_) was used to calculate the fold change of target genes [39].

## Results

### Illumina paired-end sequencing and reads assembly

**Fig. 1:**
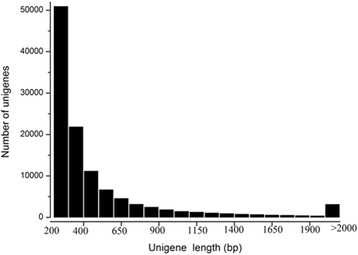
Venom apparatus transcriptome. The x-axis indicates the unigene size and Y-axis indicates the number of unigenes of each size

Sequences of mRNAs pooled from the whole body of spiderlings were analyzed by Illumina Hiseq 2000 (Illumina, USA) and generated 63 million raw reads (consisting of 6,369,225,236 bp). After removing low quality reads and adaptors, reliable reads were de novo assembled with Trinity software. A total of 159,816 transcripts were assembled ranging from 200 to 17,468 bp, with an average length size of 605 bp. Among these transcripts, 105,564 (66.05%) were smaller than 500 bp, 31,228 (19.54%) were between 500 bp and 1000 bp, 23,024 (14.41%) were longer than 1000 bp. These transcripts were further assembled and clustered. The longest sequences in each cluster were retained and designated as unigenes. Taken together, a total of 113,358 unigenes were assembled with a mean length size of 509 bp. The length distribution of assembled unigenes was shown in Fig. 1. The size distribution indicated that there were 102,465 unigenes (90.3% of the total) with the length of less than1000 bp.

### Annotation of all assembled unigenes

**Fig. 2:**
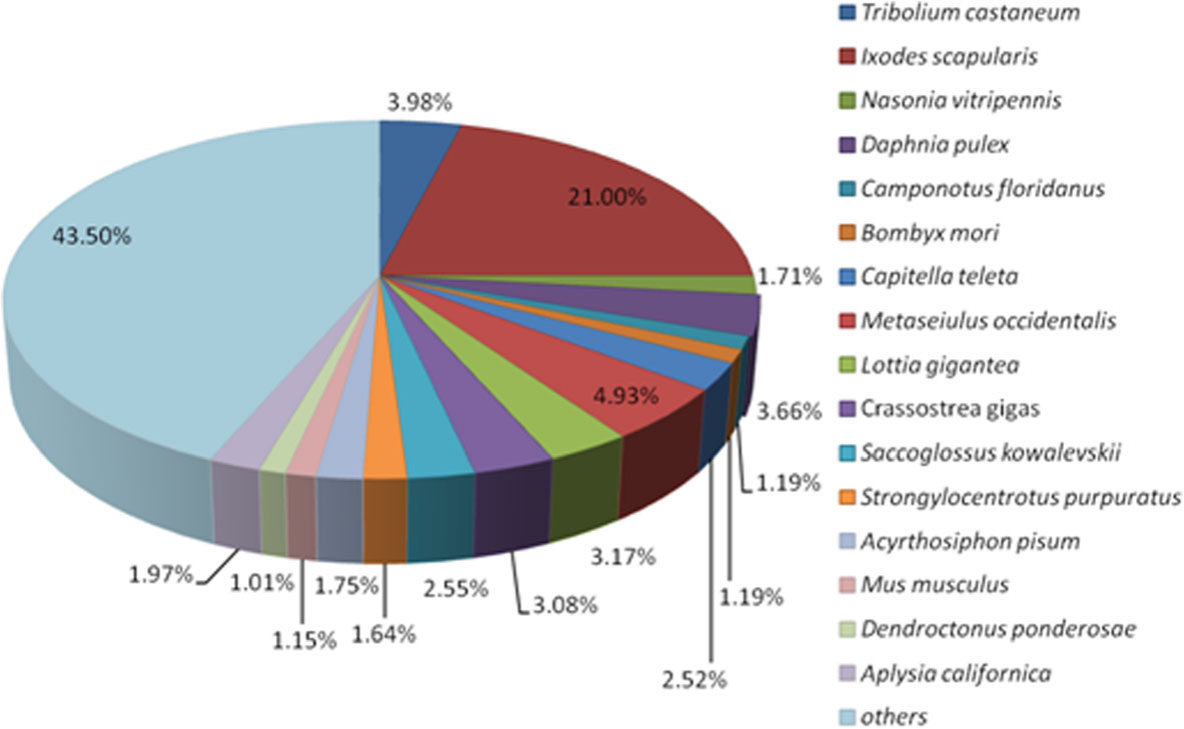
Species distribution of unigenes against Nr database. The species distribution is shown as a percentage of the total annotated sequences in NCBI Nr database with E-value < 10^−5^

The annotated unigenes were subjected to BLAST searching against Nr, Swiss-Prot, GO, KOG and KEGG databases with an E-value threshold of e < 1e^−5^. Of these, 33,509 (29.6% of all distinct sequences) unigenes could be matched Nr database (Additional file 1: Table S1), 29,042 (25.6%) to Swiss-Prot database (Additional file 2: Table S2), 28,600 (25.2%) to GO, 26,684 (23.5%) to KOG, 5521(4.9%) to KEGG. The species distribution of the top BLAST hits in Nr database for each unique sequence is shown in Fig. 2 and the summary statistics of BLAST assignment were shown in Table 1.

**Table 1:** Annotation of unigenes in venom apparatus

Database	Number of annotated unigenes	Percentage of annotated unigenes
Nr	33,509	29.6%
Swissprot	29,042	25.6%
KEGG	5521	4.9%
KOG	26,684	23.5%
GO	28,600	25.2%

All 113,358 unigenes were annotated against Nr (non-redundant), Swiss-prot, KEGG, KOG and GO databases

**Fig. 3:**
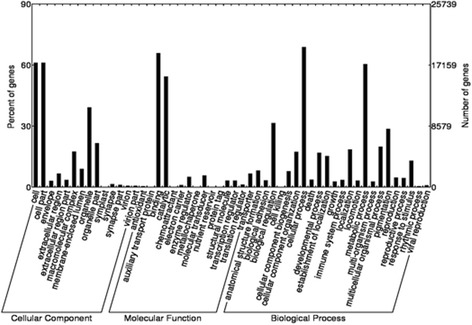
GO categories of the unigenes. 28,600 unigenes were assigned 544 GO terms, which were divided into three categories: cellular component, molecular function and biological process. The left and right Y-axis denote separately the percent and number of genes in the category

Gene ontology (GO) is dynamic ontology-based resource that provides computationally tractable and excellent broad coverage of available information about molecular biology [40]. It covers several major domains of the overall GO, which are represented by the “cellular component”, “biological process” and “molecular function”. Based on the annotation in Nr database, the annotated unigenes against GO database were collected by Blast2GO (Additional file 3: Table S3). We used WEGO software to perform GO functional classification for all the GO annotated unigenes to comprehend the distribution of gene functions of this species at the macro level (Fig. 3). Total 9973 GO term annotations corresponding to 28,600 unigenes were produced and assigned into 52 functional groups consisting of three domains: biological process (83,861), molecular function (47,010) and cellular component (27,496) (Additional file 4: Table S4).

**Fig. 4:**
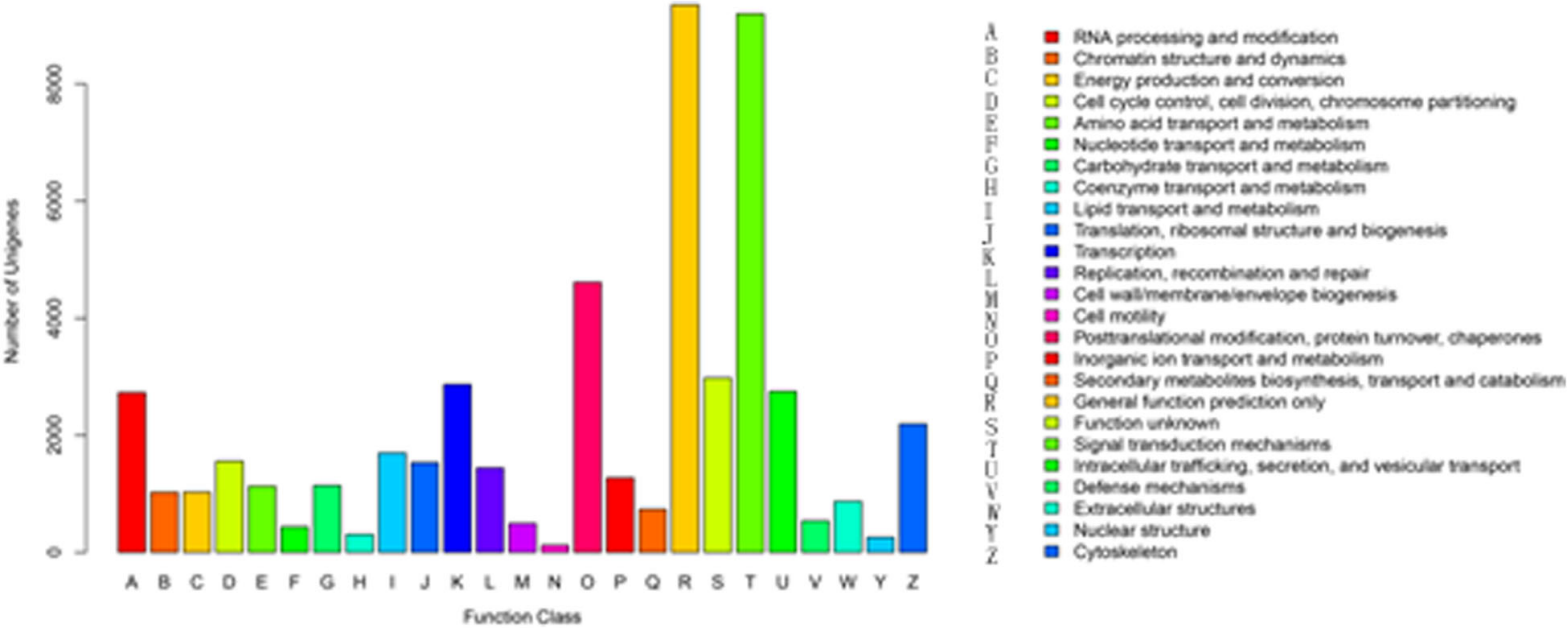
KOG classification of the unigenes. All the unigenes were aligned to the KOG database and can be classified functionally into 25 categories

To evaluate the completeness of the transcriptome library and the efficiency of annotation process, all unigenes were aligned to eukaryotic orthologous groups (KOG) database to predict and classify possible functions. From a total of 113,358 Nr hits, 26,684 unigenes (32.36% of the total) were annotated and formed 25 KOG classifications (Additional file 5: Table S5 & Fig. 4).

**Fig. 5:**
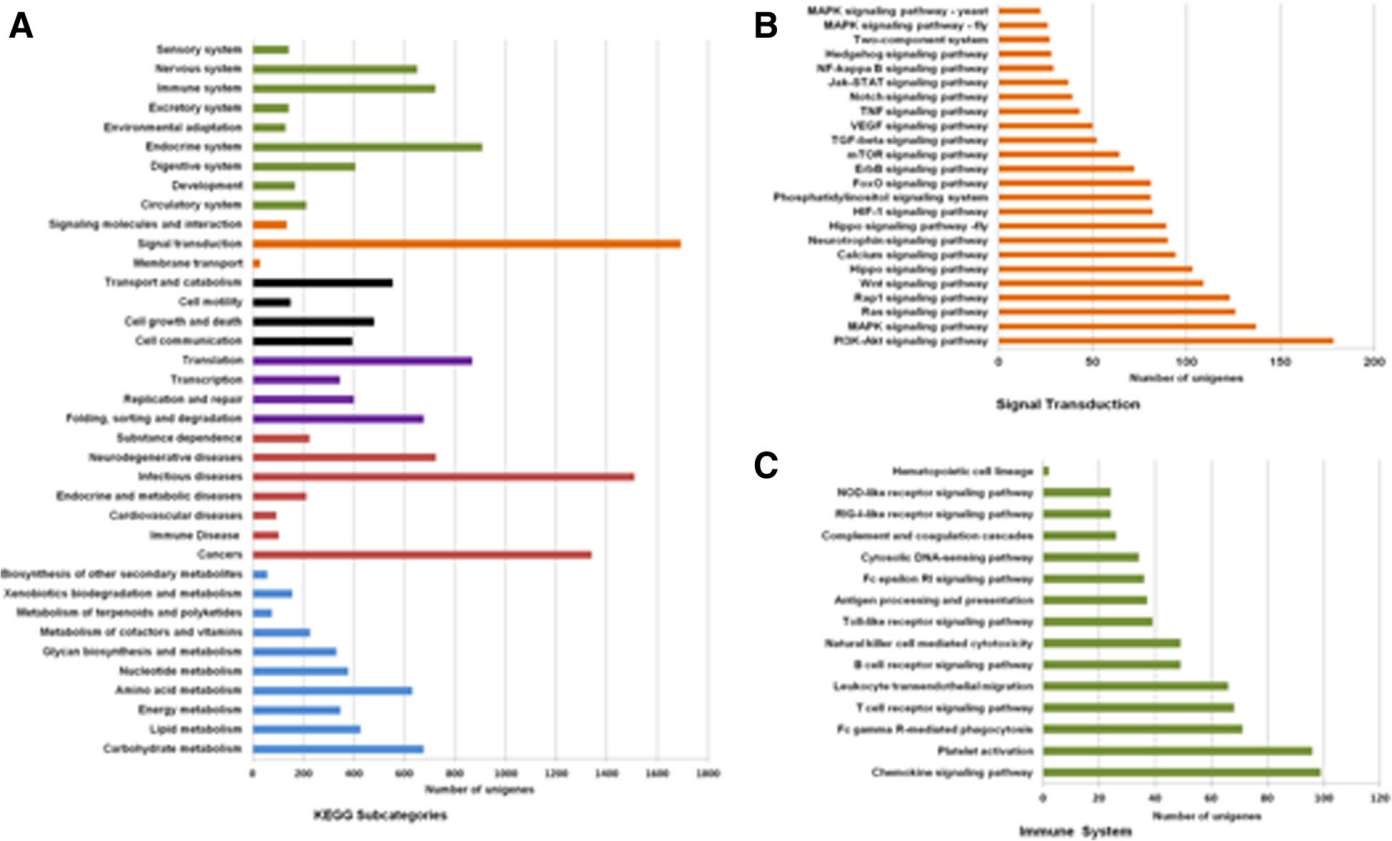
KEGG categories of annotated unigenes. **a** The unigenes grouped to different KEGG sub-categories. The number of sequences assigned to each sub-category of the six top KO categories, namely metabolism (blue), disease (red), genetic information processing (purple), cellular processes (black),environmental information processing (orange), and organismal systems (olive), were displayed. **b** Sub-categories of the unigenes grouped to signal transduction pathway; **c**: sub-categories of the unigenes grouped to immune system pathways

To identify the metabolic pathways populated by these unigenes, total 5221 annotated unigenes were grouped into six categories and mapped to 326 known metabolic or signaling pathways (Fig. 5). Among these pathways, the most representative pathways were signal transduction (1692 unigenes), infectious disease (1510 unigenes), cancers (1340 unigenes) and endocrine system (905 unigenes).

### Functional genes in response to environmental stress and immunity

In this study, the sequence and annotation information from GO and KEGG provided valuable gene resources for research on the molecular biology of these hallmarks in venom apparatus of *P. pseudoannulata.*

According to KEGG analysis, 720 unigenes were involved in 15 immune-response pathways (Fig. 5), such as ‘Chemokine signaling pathway’, ‘Platelet activation’, ‘Fc gamma R-mediated phagocytosis’, ‘Toll-like receptor signaling pathway’, ‘Complement and coagulation cascades’ and ‘NOD-like receptor signaling pathway’. A total of 1782 unigenes belong to 24 signal transduction pathways (Fig. 5). Besides the KEGG analyses, the GO annotation identified 1009 and 3726 unigenes in respond to immune system process (GO: 2,220,682) and stimulus (GO: 0050896), respectively (Additional file 6: Table S6). Heat shock protein (HSP) family performs crucial functions in correct protein folding and unfolding, translocation of proteins as well as assembly and disassembly of protein complexes. They protect hosts from stress caused by infection, inflammation or similar events [41]. As for GO annotation, the HSP families, including HSP70 and HSP90, were detected.

### Changes in gene expression in the venom apparatus

Venom, which has special function for prey capture, defense and competition deterrence, consists of a complex mixture of proteins, peptides and some small molecules. To date, little is known about how venom gland gene expression varies among species because of paucity of spider genome data. Some researchers analyzed the venom gland genome of spiders, and found the evolutionary shifts of genes among species [42, 43]^.^

To calculate the expression abundance of each gene between control and Bt-treated group, the MARS model in the DEGseq package (http://www.bioconductor.org/packages/2.6/bioc/html/DEGseq.html) was used [44]. A total of 1724 significantly changed gene entries were observed including 1654 up-regulated and 70 down-regulated genes. To identify more realistic DEGs, negative binomial distribution was introduced [45] for testifying the significant difference of reads and the value of ‘basemean’ which was used to estimate the expression abundance (Additional file 7: Figure S1). A total of 152 DEGs were observed between the two groups corresponding to 137 up-regulated and 15 down-regulated genes (Additional file 8: Table S7).

### Function annotation of DEGs

Different genes usually cooperate with each other to execute their biological functions. To comprehend the function of DEGs, we mapped all the genes with *P*-value < 0.01 to terms in the KEGG database for identifying genes involved in metabolic or signaling pathways and compared this with the whole transcriptome background. Among all the genes with KEGG annotation, 76 KEGG pathways were identified corresponding to 75 annotated genes (Additional file 9: Table S8). The main enriched metabolism pathways centered on the oxidative phosphorylation. Genetic information processing including ribosome was also enriched.

In addition, all the DEGs were also mapped to the GO terms to thoroughly describe the properties of genes and their production (Additional file 10: Table S9). A total pg 334 GO term annotations corresponding to 372 DEGs were produced and assigned into 47 functional groups and three categories. Among which 951 were assigned in biological process category, 488 in molecular function category and 826 in cellular component category (Additional file 11: Figure S2). In the category of biological process, 6 DEGs were directly associated with ‘immune system process’ (GO: 0002376) and 38 DEGs were mapped to ‘response to stimulus’ (GO: 0050896). There were annotations in several other biological processes that may indirectly participate in immune response, such as the cell cycle; DNA replication, transcription, translation; metabolism of carbohydrates, amino acid; ATPase family members, cytochrome oxidase and NADH dehydrogenase. As we discussed previously, HSP families are crucial factors in response to the environmental stress. The present study found that HSP 90 was significantly up-regulated when stressed. Considering the enriched DEGs in the venom apparatus, several important pathways and genes were explored. ATPase, cytochrome oxidase and NADH dehydrogenase were over transcribed in DEGs, indicating that a compensating of partial uncoupling oxidative phosphorylation and key role in energy metabolism.

### Validation of DEGs using qPCR

To further validate RNA-seq results, three DEGs in the venom apparatus were selected randomly for analysis by qPCR. Three DEGs were detected differentially expressed, implying that the genes expression in the venom gland of spiders is indeed affected by Bt protein.

**Fig. 6:**
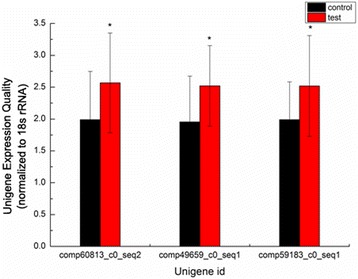
Validation of differentially expressed genes by q-pCR. Bars in each panel represent the mean ± standard error (*n* = 3), * *P* < 0.05

Unigenes comp49659_c0_ seq1, involved in defense response to bacterium, was up-regulated with 1.32-fold. Unigene comp59183_c0_seq1, participated in hormone-mediated signaling pathway, was up-regulated by 1.27-fold. Unigenes comp60183_c0_seq1, associated with organism presynaptic membrane cystine knot toxin, was up-regulated with 1.37-fold (Fig. 6). In conclusion, the validity of the sequencing data was confirmed.

## Discussion

Our previous study showed that Cry1Ab toxin expressed by transgenic rice can be trasferred and accumulated in spiders via the food web [33]. In the present study, next generation sequencing was carried out and produced 113,358 unigenes. To annotate these unigenes, distinct sequences were firstly searched against five public databases, Swiss prot, Nr, COG, GO, and KEGG with an E-value threshold of e < 1e-5. In total, 33,509 (29.6% of all distinct sequences) unigenes matched to known genes in Nr database; 29,042 (25.6% of all distinct sequences) unigenes were annotated in Swiss-Prot database. Additionally, the unigenes matched 14 of total 16 sequences from *P. pseudoannulata* and 244 of total 558 sequences from *Lycosa singoriensis* (family: Lycosidae) deposited in NCBI Nr database. The remaining unmatched unigenes (>200 bp) were further analyzed using ESTscan software and the predicted CDS were translated into peptide sequences [46].

Surprisingly, BLASTx annotation of the venom apparatus transcriptome sequences of *P. pseudoannulata* revealed the highest similarity with *I. scapularis* of the order Ixodida, and a lower identity match (4.93%) with *M. occidentalis*, a representative of the order Trombidiformes. The two orders belong to the class Arachnida, which also encompasses *P. pseudoannulata.* To expound this similarity, we compared the number of protein sequences from *I. scapularis* (5832) and *M. occidentalis* (21) (chromosome in mitochondria, 11,717 of unplaced). Clearly, the available protein sequences for *M. occidentalis* are fewer than that of *I. scapularis*, due to unavailable genome sequences for *M. occidentalis* while the genome of *I. scapularis* has already been sequenced. However, the relationship between species calls for further investigation.

Possible functions of the annotated unigenes were analyzed by matching to GO, KOG and KEGG databases. Although only a small part of unigenes were functionally annotated, the results of these searching help us to learn more properties about the venom apparatus of *P. pseudoannulata*. Interestingly, no cancer to date has ever been reported in the arthropod animal, but our results demonstrated that 1340 unigenes were matched to the ‘cancer’ KEGG pathway. Cancer is a disease of aberrant multicellularity, which is found in a multitude of living animals (from molluscs to mammals) [47, 48]. It has been reported that the core ‘multicellularity’ genes of *Amphimedon queenslandica* were implicated in cancer, indicating the ancient origins of cancer [49]. Although some unigenes of the venom apparatus of *P. pseudoannulata* were matched to the ‘cancer’ KEGG pathway, yet whether it is related to evolution of the multicellularity requires further investigation.

Invertebrate animals can be found in almost every habitat in the world. Because many of them live in environments where microorganisms thrive and proliferate, their widespread distribution and survival are largely due to the successful responses to the environmental stress and changes in immunity [50]. In addition, due to the lack of immunoglobulins, invertebrates only possess innate immunity, which is considered to be an ancient defense mechanism [51, 52]. However, the exact molecular and cellular basis of immune system remains poorly understood. In this study, the sequence and annotation information from GO and KEGG provided valuable gene resources for research on the molecular biology of these hallmarks in the venom apparatus of *P. pseudoannulata.*

Although significant progress has recently been made in studying the immune mechanism at the gene level in insects, for instance, *Apis mellifera*, *Bombyx mori*, *Drosophila melanogaster* and *Tribolium castaneum* [53–56], yet the information of immune related genes in spiders is still scarce and fragmentary. Margaret et al. isolated a 34-residue orally active insecticidal peptide (OAIP-1) from the venom of the *Australian tarantula*, which is likely to be synergized by the gut-lytic activity of Bt expressed in insect-resistant transgenic crops [57]. KEGG enrichment analyses indicated that 720 unigenes were involved in 15 immune-response pathways, including ‘Toll-like receptor signaling pathway’, ‘Complement and coagulation cascades’ and ‘NOD-like receptor signaling pathway’. The complement and coagulation systems have a fundamentally clinical role in injury and inflammation [58]. C3 (complement component 3) and C1q are the central components in complement system and were detected in this annotated pathway. Studies in the horseshoe crab have provided a breakthrough in our understanding of the coagulation pathway in arthropods [59]. It also has been reported that *I. scapularis* genome encodes for at least 11 genes that may be part of the coagulation pathway [60]. Therefore, this defense mechanism has been strongly present in arthropods. Platelets are the major regulators of coagulation and have been ascribed to an emerging role in the immune-response [61]. It has been reported that enzymes encoded by genes from the venom structures of the caterpillar *Lonomia obliqua* induced the platelet aggregation [62]. Toll like receptor signaling pathway plays a critical role in innate immunity and is highly conserved in structure and function from insects to mammals [63]. TRAF3 was detected in the database, a highly versatile regulator mediating certain innate immune receptor and cytokine receptor signals [64].

Accordingly, there were 1782 unigenes involved in 24 signal transduction pathways (Fig. 5). Among these pathways, ‘Jak-STAT signaling pathway’, ‘NF-kappa B signaling pathway’ and ‘TNF signaling pathway’ were identified and regarded as the main pathways regulating the immune response in insects [45]. SOCS (suppressor of cytokine signaling), a negative regulator of the JAK/STST pathway, was detected and exerted significant effect on regulating the immune response in *Drosophila* [65]. Nuclear factor kappa B (NF-*κ*B) transcription factors are critical to the control of response to cellular stress and are also involved in the regulation of cell-cycle/growth, survival, apoptosis, inflammation and immunity [66]. In addition, the GO annotation analyses indicated that GO terms associated with immune system process and stimulus. Among them, Heat shock protein (HSP) family performs crucial functions in correct protein folding and unfolding, translocation of proteins as well as assembly and disassembly of protein complexes. They protect hosts from stress caused by infection, inflammation or similar events [41].

Consequently, the involvement of these genes in metabolic and signaling pathways provides the basis for further identification of the biological functions of candidate genes in response to environmental stress and immunity.

Comparative transcriptome analysis indicated that some DEGs may indirectly participate in immune response, such as the cell cycle; DNA replication, transcription, translation; metabolism of carbohydrates, amino acid; ATPase family members, cytochrome oxidase and NADH dehydrogenase. As we discussed previously, HSP families are crucial factors in response to the environmental stress. The present study found that HSP 90 was significantly up-regulated when stressed. Considering the enriched DEGs in the venom apparatus, several important pathways and genes were explored. ATPase, cytochrome oxidase and NADH dehydrogenase were over transcribed in DEGs, indicating that a compensating of partial uncoupling oxidative phosphorylation and key role in energy metabolism. Besides these direct or indirect mechanisms associated with immune and stress responses, detoxification is important adaptation that allow insects to overcome the chemical defenses of plants and animals they feed on [67]. Genes related to detoxification have been identified in many species, including *A. gambiae*, *A. aegypti*, *D. melanogaster* and *A. mellifera* [68–71]. Insects considerably rely on three families of enzymes to disarm toxic xenobiotics, such as esterases (ESTs), cytochrome P450 monooxygenases (P450s) and glutathione-S-transferases (GSTs) [72]. In light of this, our study found that genes encoding GST were detected over-expressed in DEGs. In this study, we focused on the functional genes in the venom apparatus of *P. pseudoannulata* in response to environmental stress and immunity. The launching of transcriptomics study of *P. pseudoannulata*, as a non-model animal, will lay the foundation for future functional genomic research.

## Conclusions

In this study, we used high-throughput sequencing technology to identify the transcriptome of the venom apparatus of *P. pseudoannulata*, a species for which little genome data is available. The research showed that a number of candidate genes involved in immune response were identified. This transcriptome dataset will provide a valuable resource for genetic and genomic studies in *P. pseudoannulata*.

## Additional files

Additional file 1: Table S1. The unigenes annotated by BLASTx against NCBI Nr database. (XLSX 59937 kb)

Additional file 2: Table S2. The unigenes annotated by BLASTx against Swiss-Prot database. (XLSX 54995 kb)

Additional file 3: Table S3. GO annotation of unigene. (XLSX 2004 kb)

Additional file 4: Table S4. The number of annotated unigenes mapped to GO database. (XLSX 1027 kb)

Additional file 5: Table S5. KOG annotation of unigene. Annotated unigenes were categorized into 25 subgroups and listed in each sheet. (XLSX 1508 kb)

Additional file 6: Table S6. Genes involved in environmental stress and immunity based on GO annotation. (XLSX 4058 kb)

Additional file 7: Figure S1. DEGs from Bt-treated and control venom apparatus. Differential gene expression was analyzed using the DESeq package and plotted as an MA plot of log_2_ fold change versus the averages of the normalized counts. Each point represents a gene (circle) or a novel transcribed unit (triangle). Genes marked in red were detected as differentially expressed at a 1% FDR with more than a 2-fold change. (PDF 598 kb)

Additional file 8: Table S7. A list of DEGs based on negative binomial distribution. *P*-value (<0.01) and absolute value of log_2_FoldChange (>2) were used as a cut-off. (XLSX 44 kb)

Additional file 9: Table S8. Pathway enrichment analysis of DEGs. (XLSX 22 kb)

Additional file 10: Table S9. A list of DEGs annotated in GO database. (XLSX 135 kb)

Additional file 11: Figure S2. DEGs annotated in GO database by WEGO. (PDF 6 kb)

## Abbreviations

BLAST: Basic local alignment search tool
EST: Expressed sequence tag
KEGG: Kyoto encyclopedia of genes and genomes
NCBI: National center for biotechnology information
qPCR: Quantitative real-time polymerase chain reaction
