# Supplementary figures and images for: De novo characterization of venom apparatus transcriptome of Pardosa pseudoannulata and analysis of its gene expression in response to Bt protein
Source: BMC Biotechnol. 2017 Nov 7;17:73. doi: 10.1186/s12896-017-0392-z (PMC5678584; doi:10.1186/s12896-017-0392-z)

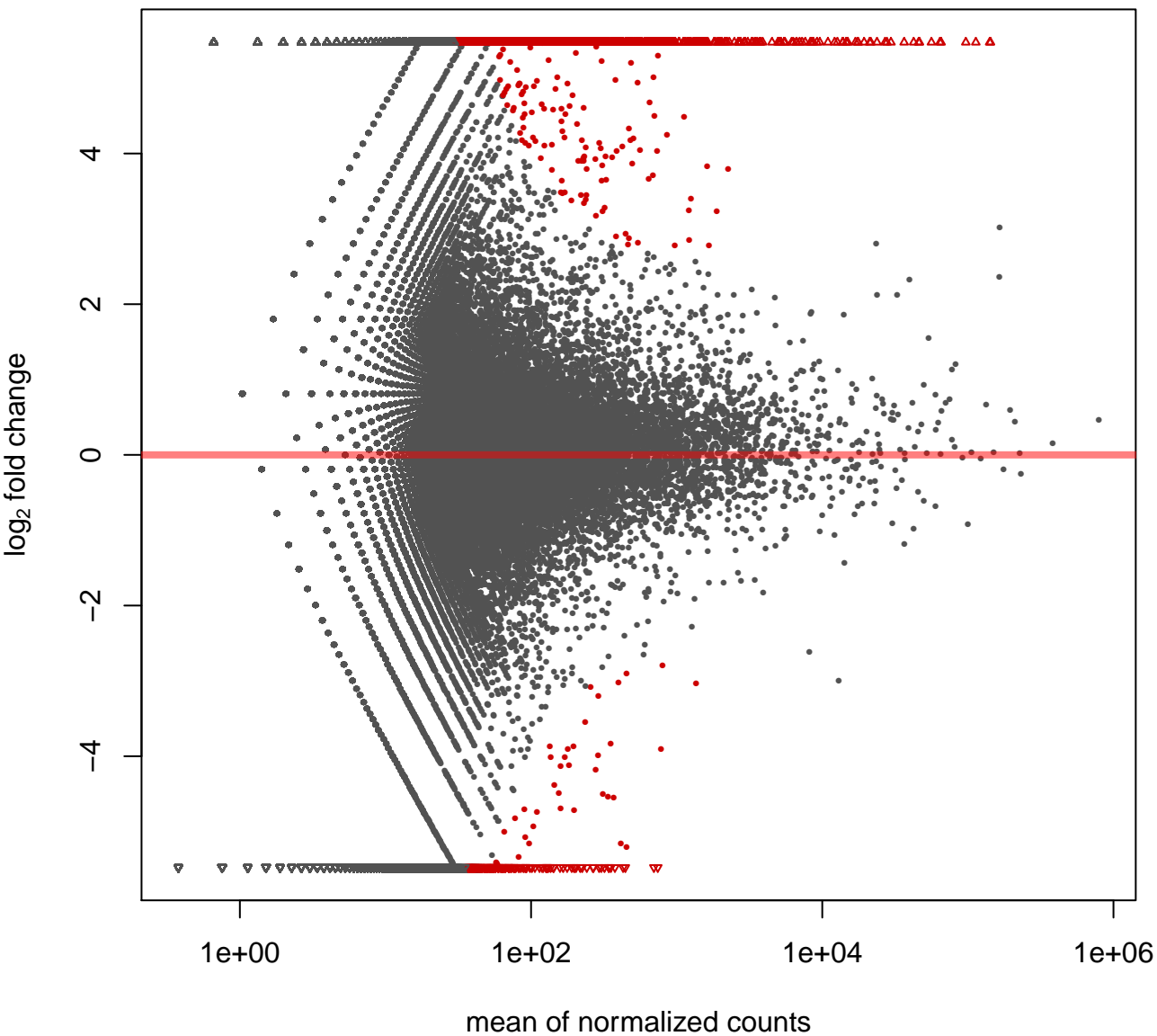

Supplement: Supplementary file 7 — DEGs from Bt-treated and control venom apparatus. Differential gene expression was analyzed using the DESeq package and plotted as an MA plot of log2 fold change versus the averages of the normalized counts. Each point represents a gene (circle) or a novel transcribed unit (triangle). Genes marked in red were detected as differentially expressed at a 1% FDR with more than a 2-fold change. (PDF 598 kb) [file 12896_2017_392_MOESM7_ESM.pdf]

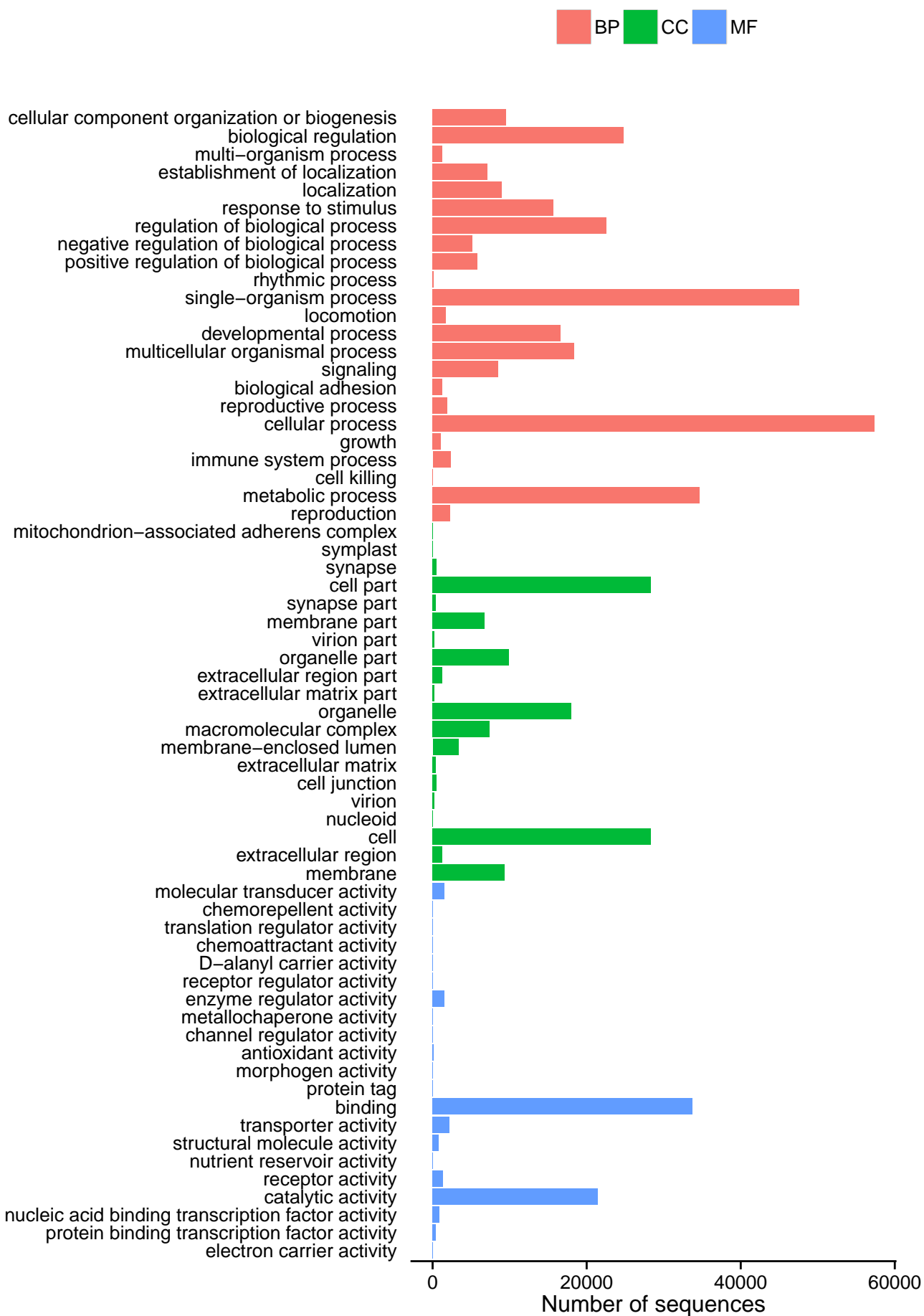

Supplement: Supplementary file 11 — DEGs annotated in GO database by WEGO. (PDF 6 kb) [file 12896_2017_392_MOESM11_ESM.pdf]
